# Data on volatile compounds produced by serotype D *Clostridium botulinum*

**DOI:** 10.1016/j.dib.2018.05.057

**Published:** 2018-05-23

**Authors:** Satoshi Nojima, Takao Myoda, Kazuki Toeda, Koichi Niwa, Toshihiro Watanabe, Yoshimasa Sagane

**Affiliations:** Department of Food and Cosmetic Science, Faculty of Bioindustry, Tokyo University of Agriculture, 196 Yasaka, Abashiri Hokkaido 099-2493, Japan

**Keywords:** TYG, trypticase peptone/yeast extract/glucose, GC, gas chromatography, MS, mass spectrometry, SPME, Solid-phase microextraction, DVB/CAR/PDMS, divinylbenzene/carboxen/polydimethylsiloxane, EI, Electron impact, *Clostridium botulinum*, Volatile compounds, Gas chromatography/mass spectrometry, Food poisoning, Botulism

## Abstract

We analyzed the volatile compounds produced by serotype D *Clostridium botulinum* (D-CB16) in trypticase peptone/yeast extract/glucose (TYG) medium using gas chromatography/mass spectrometry (GC/MS). The volatile compounds were captured by solid-phase microextraction and applied to GC/MS for separation and identification of the compounds in TYG medium with or without the cultivation of *C. botulinum* D-CB16. Thirty-five and 34 volatile compounds were identified in media without and with D-CB16 cultivation, respectively. Of the compounds identified in the medium with the strain, twenty-one were not detected in the original medium, indicating that these were produced by *C. botulinum* D-CB16.

**Specifications Table**

**Table:** 

Subject area	*Biology*
More specific subject area	*Microbiology*
Type of data	*Table*
How data was acquired	*Volatile compounds in the TYG medium with or without cultivation of C. botulinum were analyzed using gas chromatography (GC: 7890 A, Agilent Technologies, Inc.) coupled with mass spectrometry (MS: 5975 C, Agilent Technologies, Inc.).*
Data format	*Analyzed*
Experimental factors	*C. botulinum serotype D strain CB16 was cultured in trypticase peptone /yeast extract/glucose (TYG) medium.*
Experimental features	*Determined the retention index and identified the volatile compounds and their relative peak areas from the gas chromatograph.*
Data source location	*Abashiri, Japan*
Data accessibility	*Data are presented in this article*

**Value of the data**

• This is the first study to provide information on the volatile compounds produced by *C. botulinum* serotype D strain.
• The present data will help in understanding the metabolism of *C. botulinum* strains.
• The data will be useful for developing rapid detection methods for *C. botulinum* strains, which will assist in preventing outbreaks of food-borne botulism and distribution of botulinum strains related to bioterrorism.

## 1 Data

The tabular data in this article lists the retention indexes and names of the identified volatile compounds in TYG medium without or with cultivation of *Clostridium botulinum* serotype D strain CB16, as well as the relative peak area of each compound to that of the internal standard (1 µg/ml 2-octanol) in the gas chromatograph. The retention index and names of the identified compounds, as well as their relative peak areas, are provided in Table 1. The chromatograms obtained from the analyses are shown in Supplementary Figs. 1 and 2.

**Table 1:** Results of GC/MS analysis of volatile compounds collected from TYG medium without (w/o D-CB16) and with (w/ D-CB16) *C. botulinum* D-CB16 strain. The relative peak areas are indicated as ratios when the area of the internal standard (1 µl/ml 2-octanol) is set as 1.000.

Retention index	Compound	Peak area (%)	
		w/o D-CB16	w/ D-CB16
773	3-Methylbutanal	0.022 ± 0.002	n.d.
788	S-Methyl thioacetate	n.d.	0.99 ± 0.602
799	Methylcyclohexane	0.002 ± 0.000	n.d.
801	Pyrazine	0.013 ± 0.002	n.d.
807	Dimethyl disulfide	n.d.	0.345 ± 0.158
818	Methylbenzene (Toluene)	0.01 ± 0.003	n.d.
824	1,3-Dimethylcyclohexane	0.003 ± 0	n.d.
829	Butanoic acid	n.d.	0.246 ± 0.042
833	Octane	0.015 ± 0.005	n.d.
845	2-Methylpyrazine	0.051 ± 0.011	0.039 ± 0.009
847	2,6-Dimethylheptane	0.017 ± 0.001	n.d.
862	Methyl 2-Methylbutanoate	n.d.	0.003 ± 0.000
881	Xylene	0.029 ± 0.008	0.066 ± 0.017
895	1-Ethyl-4-methylcyclohexane	0.007 ± 0.001	
895	S-Methyl thiobutanoate	n.d.	0.247 ± 0.123
900	Butyl propionate	n.d.	0.32 ± 0.148
903	Nonane	0.055 ± 0.015	0.045±0.013
905	Methional	0.01 ± 0.006	n.d.
909	Benzyl propionate	n.d.	0.374 ± 0.086
929	Isopropylbenzene	0.004 ± 0.001	n.d.
957	Propylbenzene	0.005 ± 0.002	n.d.
961	Benzaldehyde	0.067 ± 0.028	0.027 ± 0.013
962	Amyl propionate	n.d.	2.049 ± 0.000
976	Dimethyl trisulfide	n.d.	0.627 ± 0.426
977	Ethyltoluene	0.01 ± 0.001	0.018 ± 0.006
991	2-Octanone	n.d.	0.044 ± 0.027
1000	Butyl butanoate	n.d.	0.353 ± 0.000
1018	2-Acetylthiazole	0.013 ± 0.003	0.026 ± 0.000
1022	2,6-Dimethylnonane	0.007 ± 0.000	n.d.
1033	*dl*-Limonene	0.007 ± 0.003	0.012 ± 0.004
1041	Butyl 2-methylbutanoate	n.d.	0.286 ± 0.043
1045	Benzeneacetaldehyde	0.018 ± 0.009	n.d.
1055	Isoamyl butanoate	n.d.	0.666 ± 0.506
1064	2-Methyldecane	0.004 ± 0.001	n.d.
1068	Acetophenone	0.005 ± 0.001	0.015 ± 0.009
1079	3-Ethyl-2,5-dimethylpyrazine	0.011 ± 0.003	n.d.
1083	1-Ethyl-2,3-dimethylbenzene	0.003 ± 0.002	n.d.
1092	2-Nonanone	n.d.	0.565 ± 0.408
1101	Undecane	0.027 ± 0.009	n.d.
1104	Nonanal	0.017 ± 0.008	n.d.
1115	Phenylethyl alcohol	n.d.	0.034 ± 0.031
1119	2-Formyl-5-methylthiophene	0.027 ± 0.009	0.039 ± 0.017
1122	3-Methyl-2-thiophenecarboxaldehyde	0.054 ± 0.021	n.d.
1152	3-Methylbutyl pentanoate	n.d.	0.054 ± 0.04
1177	*l*-Menthol	n.d.	0.012 ± 0.001
1193	2-Decanone	n.d.	0.073 ± 0.053
1201	Dodecane	0.015 ± 0.005	0.025 ± 0.008
1206	Decanal	0.009 ± 0.005	0.011 ± 0.007
1213	2,6-Dimethylundecane	0.002 ± 0.001	n.d.
1223	Dimethyl tetrasulfide	n.d.	0.09 ± 0.084
1225	3-Phenylfuran	0.006 ± 0.001	0.011 ± 0.008
1258	Benzyl propionate	n.d.	0.007 ± 0.004
1294	Indole	0.006 ± 0.002	2.899 ± 1.831
1300	Tridecane	0.014 ± 0.006	n.d.
1400	Tetradecane	0.008 ± 0.004	n.d.
1433	β-Phenylethyl butanoate	n.d.	0.045 ± 0.028

n.d.: not detected

## 2 Experimental design, materials and methods

### 2.1 Design

Serotype D of *C. botulinum* strain CB16 (D-CB16) was cultured in TYG medium for production of volatile compounds. Volatile compounds were captured from medium both with and without cultivation of the strain.

### 2.2 Materials

*C. botulinum* D-CB16, obtained from Japanese soil [1], was used in this study.

### 2.3 Cultivation of C. botulinum strains

*C. botulinum* D-CB16 was precultured at 37°C overnight in 50 ml TYG medium (pH 7.2), which contained 3% trypticase peptone (BD Biosciences, Franklin Lakes, NJ, USA), 2% yeast extract (BD Biosciences), 0.5% glucose (Wako Pure Chemical, Osaka, Japan), and 0.15% cysteine-HCl (Wako Pure Chemical), as previously reported [2]. The preculture was inoculated in 2500 ml fresh TYG medium, and then further cultured at 37 °C overnight. Three thousand milliliters fresh TYG medium was used as “medium without cultivation of the strain.” As an internal standard, 1 µl/ml 2-octanol (10 µl) was added to each medium.

### 2.4 Volatile compound analysis

Solid-phase microextraction (SPME) was used to identify the volatile compounds in the medium. Volatile compounds in the samples were extracted using a SPME fiber coated with 50/30 µm divinylbenzene/carboxen/polydimethylsiloxane (DVB/CAR/PDMS) (Supelco Co., Bellefonte, PA, USA) with 1-cm standard needle for manual operation Supelco Co., Ref. 57328-U, Bellefonte, PA, USA. Briefly, the SPME fiber was exposed to the headspace of 3,000 mL of each medium at 37 °C for 1 h to capture the volatile compounds, following which, it was injected into a gas chromatography (GC) (7890 A, Agilent Technologies Inc., Santa Clara, CA. USA) DB-5 column (60 m × 0.32 mm i.d., 0.25 μm film thickness; Agilent Technologies Inc.) coupled with mass spectrometry (MS) (5975 C, Agilent Technologies, Inc.) at 220 °C for 5 min in splitless mode. The oven temperature was initially held at 60 °C and then increased to 230°C at the rate of 3°C/min. Helium was used as the carrier gas at a flow rate of 2.0 mL/min. The temperature of the detector was held at 230°C. Electron impact (EI) mass spectra were recorded at 70 eV in an *m*/*z* range of 30–400. The compounds were identified by their GC retention indices, which were calculated from their retention time with respect to those of a series of C_6_-C_18_ *n*-alkanes on a DB-5 capillary column (30 m × 0.25 mm i.d., 0.25 film thickness; J & W Scientific), and by computer matching using AromaOffice 2D (Nishikawa Keisoku, Tokyo, Japan). The relative amount of each compound was determined from the respective peak area compared to that of 1 µl/ml 2-octanol.

## Appendix A Supplementary material

Supplementary figure 1. Chromatogram on the GC analysis of volatile compounds obtained from TYG medium with *C. botulinum* D-CB16 strain.Supplementary material

.

Supplementary figure 2. Chromatogram on the GC analysis of volatile compounds obtained from TYG medium without *C. botulinum* D-CB16 strain.Supplementary material

## References

[1] Oguma K., Yamaguchi T., Sudou K., Yokosawa N., Fujikawa Y. (1986). Biochemical classification of Clostridium botulinum type C and D strains and their nontoxigenic derivatives. Appl. Environ. Microbiol.

[2] Sagane Y., Hasegawa K., Mutoh S., Kouguchi H., Suzuki T., Sunagawa H., Nakagawa T., Kamaguchi A., Okasaki S., Nakayama K., Watanabe T., Oguma K., Ohyama T. (2003). Molecular characterization of GroES and GroEL homologues from Clostridium botulinum. J. Protein Chem..

