# Supplementary figures and images for: Data on volatile compounds produced by serotype D Clostridium botulinum
Source: Data Brief. 2018 May 23;19:393–7. doi: 10.1016/j.dib.2018.05.057 (PMC5997912; doi:10.1016/j.dib.2018.05.057)

Supplementary figure 1

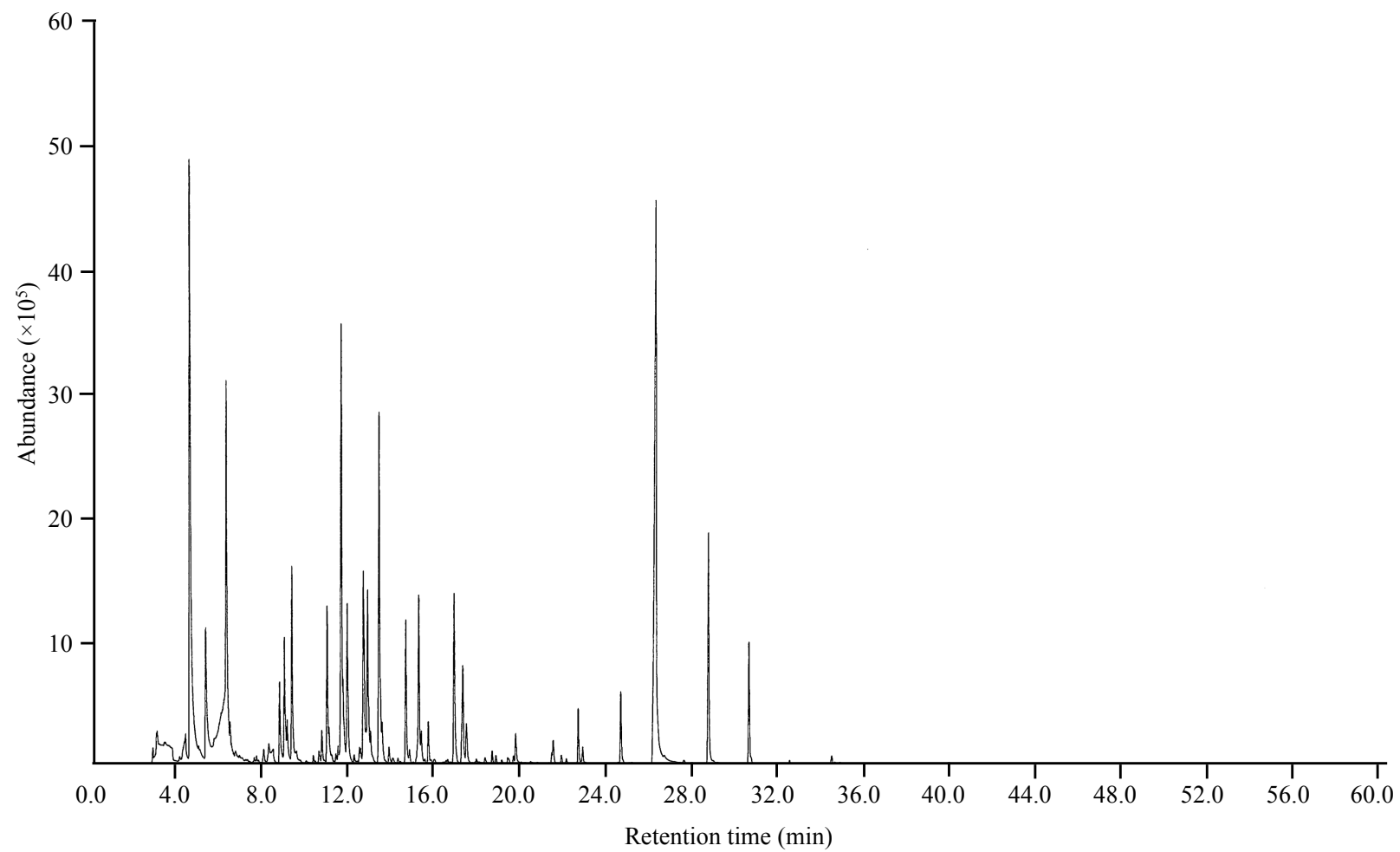

Supplement: Supplementary file 2 — Supplementary figure 1. Chromatogram on the GC analysis of volatile compounds obtained from TYG medium with C. botulinum D-CB16 strain.Supplementary material [file mmc2.pdf]

Supplementary figure 2

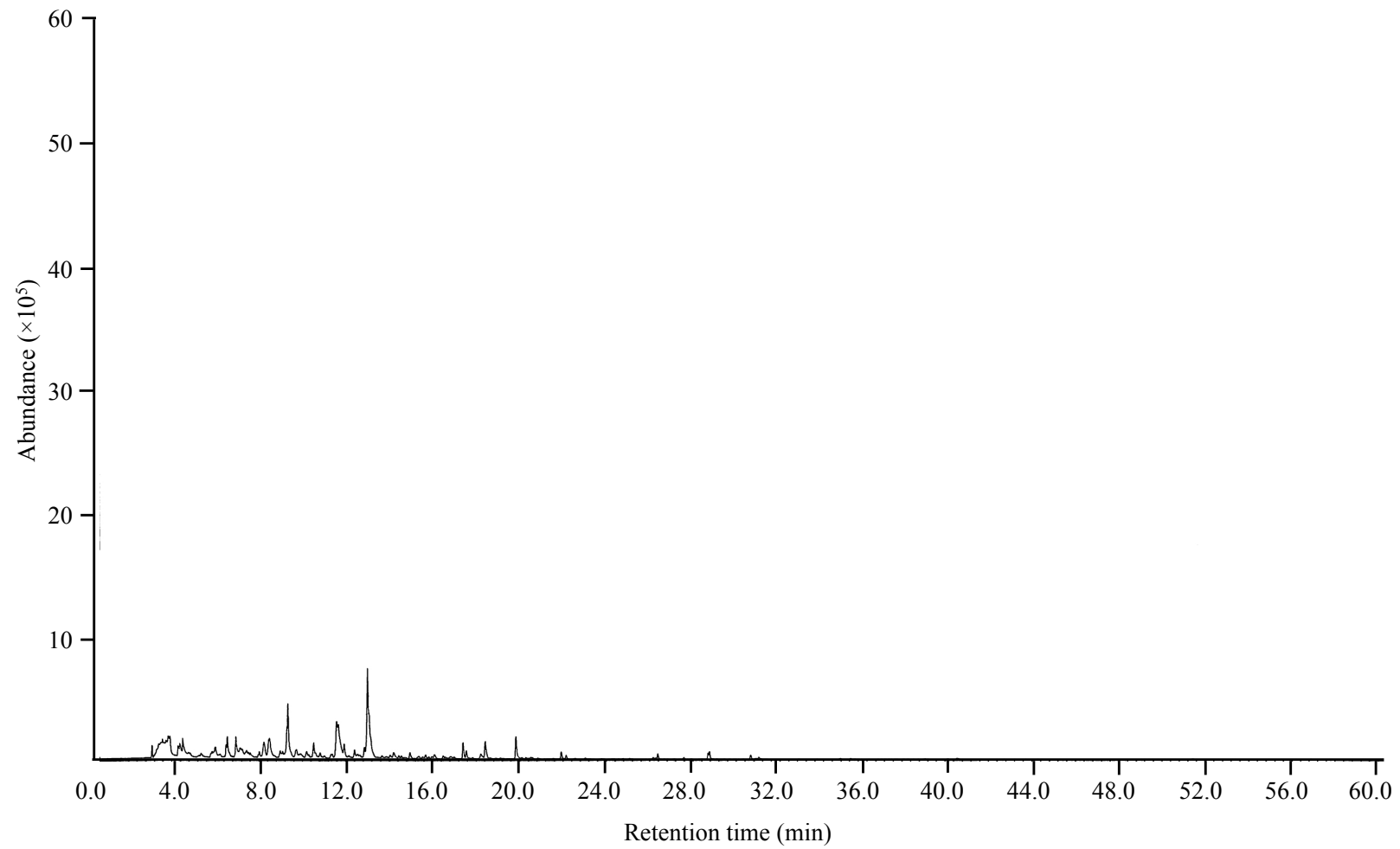

Supplement: Supplementary file 3 — Supplementary figure 2. Chromatogram on the GC analysis of volatile compounds obtained from TYG medium without C. botulinum D-CB16 strain.Supplementary material [file mmc3.pdf]
